# Supplementary material for: Linking muscle mechanics to the metabolic cost of human hopping
Source: J Exp Biol. 2023 Jun 15;226(12):jeb245614. doi: 10.1242/jeb.245614 (PMC10281547; doi:10.1242/jeb.245614)
Supplement: Supplementary information [file jexbio-226-245614-s1.pdf]

Journal of Experimental Biology: doi:10.1242/jeb.245614: Supplementary information

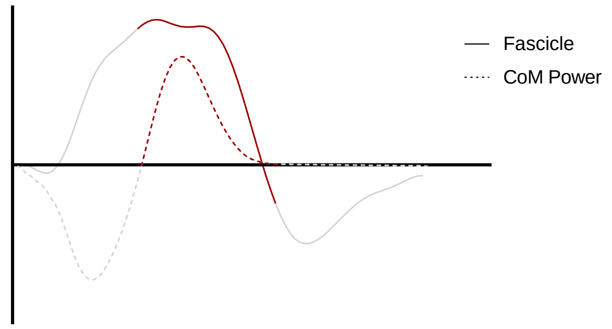

Fig. S1. Example corresponding fascicle length and CoM power time-series data. The period of positive CoM power is indicated in red, and was used in our calculations of fascicle shortening, mean fascicle shortening velocity, and fascicle to MTU shortening ratio.

Journal of Experimental Biology • Supplementary information

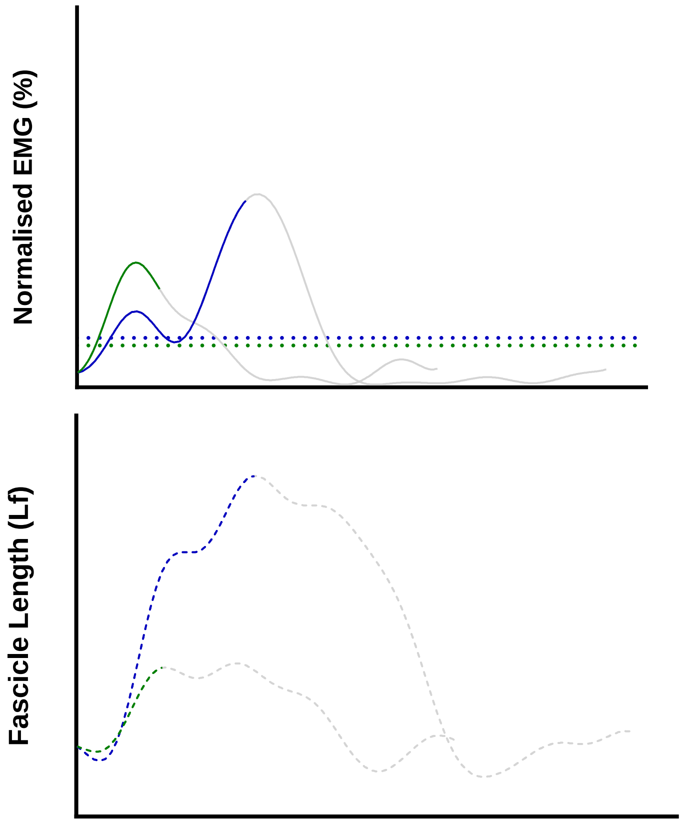

Fig. S2. Example corresponding normalised SOL muscle activity and fascicle length time-series data from two conditions that differ in the amount of eccentric work performed by muscle. The coloured, non-grey, portions of the data represent the period of fascicle lengthening. The condition in blue, which had more fascicle lengthening than the condition in green, had a similar mean level of activation during this eccentric phase (0.20% and 0.22%, respectively). Both conditions had a similar mean level of activation over the entire hop cycle (0.13% and 0.10%, respectively (shown by the two dotted horizontal lines)).
